# Supplementary material for: Viral kinetics of sequential SARS-CoV-2 infections
Source: Nat Commun. 2023 Oct 5;14:6206. doi: 10.1038/s41467-023-41941-z (PMC10556125; doi:10.1038/s41467-023-41941-z)
Supplement: Supplementary file 1 — Supplementary Information [file 41467_2023_41941_MOESM1_ESM.pdf]

Supplementary Table 1. Characteristics of the well-documented first infections. Counts by lineage, vaccination status, and age group for the 1,796 well-documented infections. Well-documented infections are those with at least one RT-qPCR Ct < 32 and three Ct < 40 (the limit of detection). Infections occurred between 11th March 2020 and 28th July 2022.

Statistic	Category	N	%
**Total**		**1796**	**100**
**Lineage + vaccination status at time of infection**	**Alpha**	**48**	**2.7**
	Unvaccinated	23	1.3
	Vaccinated	4	0.2
	Unknown primary vaccination status	21	1.2
	Unboosted	22	1.2
	Boosted	0	0.0
	Unknown booster status	26	1.4
	**Delta**	**191**	**10.6**
	Unvaccinated	7	0.4
	Vaccinated	123	6.8
	Unknown primary vaccination status	61	3.4
	Unboosted	99	5.5
	Boosted	10	0.6
	Unknown booster status	82	4.6
	**BA.1/BA.2**	**1241**	**69.1**
	Unvaccinated	4	0.2
	Vaccinated	855	47.6
	Unknown primary vaccination status	382	21.3
	Unboosted	87	4.8
	Boosted	699	38.9
	Unknown booster status	455	25.3
	**BA.4/BA.5**	**61**	**3.4**
	Unvaccinated	0	0.0
	Vaccinated	47	2.6
	Unknown primary vaccination status	14	0.8
	Unboosted	0	0.0
	Boosted	44	2.4
	Unknown booster status	17	0.9
	**Other/Unspecified**	**255**	**14.2**
	Unvaccinated	93	5.2
	Vaccinated	66	3.7
	Unknown primary vaccination status	96	5.3
	Unboosted	87	4.8
	Boosted	43	2.4
	Unknown booster status	125	7
**Age group**	0-29	711	39.6
	30-49	795	44.3
	50-99	288	16.0
	Not Reported	2	0.1

Supplementary Table 2. Characteristics of the well-documented second infections. Counts by lineage, vaccination status, and age group for the 193 well-documented second infections. Well-documented infections are those with at least one RT-qPCR Ct < 32 and three Ct < 40 (the limit of detection). Infections occurred between 11th March 2020 and 28th July 2022.

Statistic	Category	N	%
**Total**		**193**	**100**
**Lineage + vaccination status at time of infection**	**Alpha**	**1**	**0.5**
	Unvaccinated	1	0.5
	Vaccinated	0	0
	Unknown primary vaccination status	0	0
	Unboosted	0	0
	Boosted	0	0
	Unknown booster status	1	0.5
	**Delta**	**0**	**0**
	Unvaccinated	0	0
	Vaccinated	0	0
	Unknown primary vaccination status	0	0
	Unboosted	0	0
	Boosted	0	0
	Unknown booster status	0	0
	**BA.1/BA.2**	**159**	**82.4**
	Unvaccinated	2	1
	Vaccinated	139	72.0
	Unknown primary vaccination status	18	9.3
	Unboosted	30	15.5
	Boosted	79	40.9
	Unknown booster status	50	25.9
	**BA.4/BA.5**	**10**	**5.2**
	Unvaccinated	0	0
	Vaccinated	6	3.1
	Unknown primary vaccination status	4	2.1
	Unboosted	0	0
	Boosted	6	3.1
	Unknown booster status	4	2.1
	**Other/Unspecified**	**23**	**11.9**
	Unvaccinated	5	2.6
	Vaccinated	15	7.8
	Unknown primary vaccination status	3	1.6
	Unboosted	5	2.6
	Boosted	12	6.2
	Unknown booster status	6	3.1
**Age group**	0-29	105	54.4
	30-49	81	42.0
	50-99	7	3.6
	Not Reported	0	0

Supplementary Table 3. Viral kinetic parameters for first and second infections in the 71 individuals with two well-documented infections. Values represent posterior means with 95% credible intervals in parentheses.

**Viral kinetic parameter**	**Category**	**Value (95% CI)**
Peak viral concentration (Ct)	First Infection	25.9 (24.6, 27.1)
	Second Infection	27.4 (25.9, 28.7)
Peak viral concentration (GE/ml)	First Infection	6.6 (6.2, 6.9)
	Second Infection	6.2 (5.8, 6.6)
Proliferation time (days)	First Infection	4.5 (3.5, 5.8)
	Second Infection	4.0 (2.9, 5.4)
Clearance time (days)	First Infection	9.2 (8.1, 10.3)
	Second Infection	6.3 (5.3, 7.4)
Proliferation rate (Ct/day)	First Infection	3.2 (2.4, 4.1)
	Second Infection	3.2 (2.2, 4.5)
Clearance rate (Ct/day)	First Infection	1.5 (1.3, 1.8)
	Second Infection	2.0 (1.6, 2.5)

Supplementary Table 4. Viral kinetic parameters for all well-documented first (n = 1,796) and second (n = 193) infections. Values represent posterior means with 95% credible intervals in parentheses.

Viral kinetic parameter	Category	Value (95% CI)
Peak viral concentration (Ct)	First Infection	25.7 (24.6, 26.7)
	Second Infection	26.9 (25.7, 28)
Peak viral concentration (GE/ml)	First Infection	6.6 (6.4, 6.9)
	Second Infection	6.3 (6.0, 6.6)
Proliferation time (days)	First Infection	4.9 (4.1, 5.8)
	Second Infection	4.1 (3.2, 5.2)
Clearance time (days)	First Infection	9.3 (8.5, 10.2)
	Second Infection	6.6 (5.8, 7.3)
Proliferation rate (Ct/day)	First Infection	3.0 (2.4, 3.6)
	Second Infection	3.3 (2.5, 4.2)
Clearance rate (Ct/day)	First Infection	1.5 (1.4, 1.7)
	Second Infection	2.0 (1.7, 2.3)

Supplementary Table 5. Viral kinetic parameters for second infections in the 71 individuals with two well-documented infections, stratified by vaccination status. Vaccination statuses are marked from the recorded time of infection and include not vaccinated/boost unknown (n=1), vaccinated/not boosted (n=10), vaccinated/boosted (n=43), vaccinated/boost unknown (n=7), and fully unknown (n=10). Values represent posterior means with 95% credible intervals in parentheses.

Viral kinetic parameter	Category	Value (95% CI)
Peak viral concentration (Ct)	Not vaccinated/Boost unknown	28.6 (20.2, 34.2)
	Vaccinated/Not boosted	27.7 (24.2, 30.7)
	Vaccinated/Boosted	30.0 (28.0, 31.8)
	Vaccinated/Boost unknown	31.1 (27.8, 33.7)
	Unknown	28.7 (25.6, 31.5)
Peak viral concentration (GE/ml)	Not vaccinated/Boost unknown	5.8 (4.3, 8.2)
	Vaccinated/Not boosted	6.1 (5.2, 7.0)
	Vaccinated/Boosted	5.4 (4.9, 6.0)
	Vaccinated/Boost unknown	5.1 (4.4, 6.0)
	Unknown	5.8 (5.0, 6.6)
Proliferation time (days)	Not vaccinated/Boost unknown	6.1 (1.6, 18.7)
	Vaccinated/Not boosted	4.1 (2.0, 7.9)
	Vaccinated/Boosted	4.7 (2.5, 8.1)
	Vaccinated/Boost unknown	6.9 (2.2, 17.2)
	Unknown	4.5 (2.1, 8.6)
Clearance time (days)	Not vaccinated/Boost unknown	5.5 (1.8, 12.9)
	Vaccinated/Not boosted	5.4 (3.4, 8.1)
	Vaccinated/Boosted	5.9 (4.3, 8.0)
	Vaccinated/Boost unknown	4.6 (2.5, 7.6)
	Unknown	5.7 (3.6, 8.9)
Proliferation rate (Ct/day)	Not vaccinated/Boost unknown	2.9 (0.5, 8.7)
	Vaccinated/Not boosted	3.3 (1.4, 6.7)
	Vaccinated/Boosted	2.3 (1.1, 4.3)
	Vaccinated/Boost unknown	1.7 (0.4, 4.3)
	Unknown	2.9 (1.1, 5.9)
Clearance rate (Ct/day)	Not vaccinated/Boost unknown	2.7 (0.7, 6.8)
	Vaccinated/Not boosted	2.4 (1.4, 3.8)
	Vaccinated/Boosted	1.7 (1.2, 2.5)
	Vaccinated/Boost unknown	2.1 (1.1, 3.8)
	Unknown	2.1 (1.1, 3.3)

Supplementary Table 6. Viral kinetic parameters for second infections in the 193 well-documented second infections. Vaccination statuses are marked from the recorded time of infection and include not vaccinated/not boosted (n=2), not vaccinated/boost unknown (n=6), vaccinated/not boosted (n=33), vaccinated/boosted (n=97), vaccinated/boost unknown (n=30), and fully unknown (n=25). Values represent posterior means with 95% credible intervals in parentheses.

Viral kinetic parameter	Category	Value (95% CI)
Peak viral concentration (Ct)	Not vaccinated/not boosted	26.4 (21.1, 30.8)
	Not vaccinated/Boost unknown	30.5 (27.2, 33.3)
	Vaccinated/Not boosted	28.8 (26.5, 30.9)
	Vaccinated/Boosted	29.2 (27.2, 31.1)
	Vaccinated/Boost unknown	28.8 (26.3, 31.2)
	Unknown	28.4 (25.8, 30.8)
Peak viral concentration (GE/ml)	Not vaccinated/not boosted	6.4 (5.2, 7.9)
	Not vaccinated/Boost unknown	5.3 (4.5, 6.2)
	Vaccinated/Not boosted	5.8 (5.2, 6.4)
	Vaccinated/Boosted	5.6 (5.1, 6.2)
	Vaccinated/Boost unknown	5.8 (5.1, 6.5)
	Unknown	5.9 (5.2, 6.6)
Proliferation time (days)	Not vaccinated/not boosted	4.8 (2.3, 9.1)
	Not vaccinated/Boost unknown	9 (3.4, 20.9)
	Vaccinated/Not boosted	3.9 (2.2, 6.4)
	Vaccinated/Boosted	5.5 (3.1, 8.7)
	Vaccinated/Boost unknown	4.0 (2.2, 6.6)
	Unknown	5.6 (2.8, 9.8)
Clearance time (days)	Not vaccinated/not boosted	9.1 (5.3, 14.3)
	Not vaccinated/Boost unknown	5.5 (3.3, 8.8)
	Vaccinated/Not boosted	4.8 (3.5, 6.4)
	Vaccinated/Boosted	5.7 (4.3, 7.4)
	Vaccinated/Boost unknown	5.3 (3.8, 7.1)
	Unknown	5.8 (4.1, 7.9)
Proliferation rate (Ct/day)	Not vaccinated/not boosted	3.2 (1.4, 6.6)
	Not vaccinated/Boost unknown	1.3 (0.4, 3.2)
	Vaccinated/Not boosted	3.1 (1.7, 5.5)
	Vaccinated/Boosted	2.1 (1.1, 3.7)
	Vaccinated/Boost unknown	3.0 (1.5, 5.4)
	Unknown	2.3 (1.1, 4.4)
Clearance rate (Ct/day)	Not vaccinated/not boosted	1.6 (0.8, 2.8)
	Not vaccinated/Boost unknown	1.8 (1.0, 3.1)
	Vaccinated/Not boosted	2.4 (1.6, 3.4)
	Vaccinated/Boosted	1.9 (1.3, 2.6)
	Vaccinated/Boost unknown	2.2 (1.4, 3.1)
	Unknown	2.1 (1.4, 3.0)

Supplementary Table 7. Viral kinetic parameters for the 71 second infections in individuals with two well-documented infections, stratified by the variant category of the prior infection. Prior infections were caused by Other/None (n=36), Alpha (n=13), Delta (n=16), or BA.1/BA.2 (n=6) lineages. Values represent posterior means with 95% credible intervals in parentheses.

Viral kinetic parameter	Category	Value (95% CI)
Peak viral concentration (Ct)	Other/None	27.5 (22.2, 31.8)
	Alpha	27 (21.1, 31.8)
	Delta	26.9 (20.9, 31.7)
	BA.1/BA.2	27.1 (20.4, 31.8)
Peak viral concentration (GE/ml)	Other/None	6.1 (4.9, 7.6)
	Alpha	6.3 (4.9, 7.9)
	Delta	6.3 (5.0, 8.0)
	BA.1/BA.2	6.2 (4.9, 8.1)
Proliferation time (days)	Other/None	4.3 (1.8, 8.7)
	Alpha	5.7 (2.3, 12.0)
	Delta	4.3 (1.7, 9.0)
	BA.1/BA.2	4.4 (1.3, 10.6)
Clearance time (days)	Other/None	8.5 (4.6, 14.2)
	Alpha	10.4 (5.8, 17.6)
	Delta	8.4 (4.2, 14.6)
	BA.1/BA.2	5.7 (2.4, 11.4)
Proliferation rate (Ct/day)	Other/None	3.5 (1.2, 7.5)
	Alpha	2.8 (0.9, 6.3)
	Delta	3.6 (1.2, 8.5)
	BA.1/BA.2	4.0 (1.0, 10.9)
Clearance rate (Ct/day)	Other/None	1.6 (0.8, 3.0)
	Alpha	1.4 (0.6, 2.6)
	Delta	1.7 (0.8, 3.5)
	BA.1/BA.2	2.7 (1.0, 5.8)

Supplementary Table 8. Viral kinetic parameters for the 193 well-documented second infections stratified by the variant category of the prior infection. Prior infections were caused by Other/None (n=146), Alpha (n=16), Delta (n=23), or BA.1/BA.2 (n=8) lineages. Values represent posterior means with 95% credible intervals in parentheses.

**Viral kinetic parameter**	**Category**	**Value (95% CI)**
Peak viral concentration (Ct)	Other/None	26.5 (20.6, 30.6)
	Alpha	26.7 (20.5, 31.4)
	Delta	27 (20.7, 31.1)
	BA.1/BA.2	26.3 (18.9, 31.6)
Peak viral concentration (GE/ml)	Other/None	6.4 (5.2, 8)
	Alpha	6.3 (5, 8.1)
	Delta	6.3 (5.1, 8)
	BA.1/BA.2	6.4 (5, 8.5)
Proliferation time (days)	Other/None	4.7 (2.2, 8.8)
	Alpha	5.5 (2.1, 11.3)
	Delta	4.9 (2.0, 10)
	BA.1/BA.2	4.4 (1.4, 10.8)
Clearance time (days)	Other/None	9.1 (5.2, 14.8)
	Alpha	9.8 (5.2, 17.0)
	Delta	8.9 (4.7, 14.7)
	BA.1/BA.2	6.4 (2.9, 12.2)
Proliferation rate (Ct/day)	Other/None	3.2 (1.3, 6.7)
	Alpha	2.9 (1.0, 6.5)
	Delta	3.2 (1.1, 7.3)
	BA.1/BA.2	4.0 (1.0, 11)
Clearance rate (Ct/day)	Other/None	1.6 (0.8, 2.8)
	Alpha	1.5 (0.7, 2.8)
	Delta	1.6 (0.7, 3.1)
	BA.1/BA.2	2.4 (1.0, 5.0)

Supplementary Table 9. Correlation coefficients between adjusted posterior first- and second-infection viral kinetic parameters for the 71 individuals with two well-documented infections. Values represent posterior means with 95% credible intervals in parentheses.

Correlation type	Viral kinetic parameter	Correlation coefficient
Pearson	Peak viral concentration (Ct)	0.06 (-0.11, 0.25)
	Proliferation time (days)	0.10 (-0.12, 0.34)
	Clearance time (days)	0.26 (0.09, 0.43)
Spearman	Peak viral concentration (Ct)	0.08 (-0.10, 0.26)
	Proliferation time (days)	0.12 (-0.09, 0.33)
	Clearance time (days)	0.30 (0.12, 0.46)

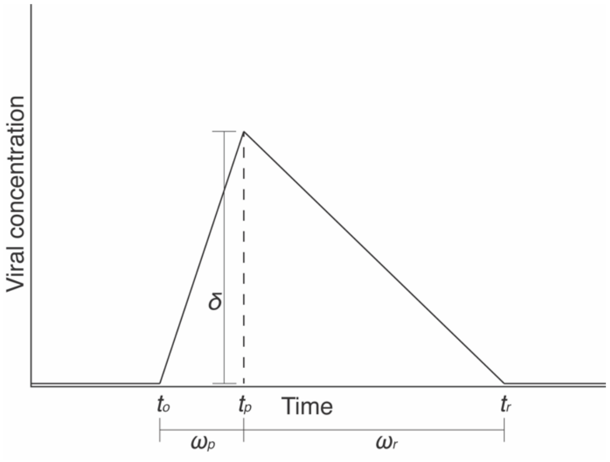

Supplementary Figure 1. Schematic viral concentration profile for an acute infection. According to the viral kinetic model, the $\log_2$ viral concentration begins at the limit of detection, then increases linearly starting at time to t_o a peak of δ at time t_p. Then, the $\log_2$ viral concentration decays linearly back to the limit of detection, arriving there at time t_r. From these values, we can derive the proliferation time ω_p and the clearance time ω_r.
