# Supplementary material for: Viral kinetics of sequential SARS-CoV-2 infections
Source: Nat Commun. 2023 Oct 5;14:6206. doi: 10.1038/s41467-023-41941-z (PMC10556125; doi:10.1038/s41467-023-41941-z)
Supplement: Supplementary file 3 — Reporting Summary [file 41467_2023_41941_MOESM3_ESM.pdf]

nature portfolio

Corresponding author(s): Yonatan Grad

Last updated by author(s): August 8, 2023

Reporting Summary

Nature Portfolio wishes to improve the reproducibility of the work that we publish. This form provides structure for consistency and transparency in reporting. For further information on Nature Portfolio policies, see our Editorial Policies and the Editorial Policy Checklist.

Statistics

For all statistical analyses, confirm that the following items are present in the figure legend, table legend, main text, or Methods section.

n/a	Confirmed	
☐	☒	The exact sample size (*n*) for each experimental group/condition, given as a discrete number and unit of measurement
☐	☒	A statement on whether measurements were taken from distinct samples or whether the same sample was measured repeatedly
☐	☒	The statistical test(s) used AND whether they are one- or two-sided<br>*Only common tests should be described solely by name; describe more complex techniques in the Methods section.*
☐	☒	A description of all covariates tested
☐	☒	A description of any assumptions or corrections, such as tests of normality and adjustment for multiple comparisons
☐	☒	A full description of the statistical parameters including central tendency (e.g. means) or other basic estimates (e.g. regression coefficient) AND variation (e.g. standard deviation) or associated estimates of uncertainty (e.g. confidence intervals)
☐	☒	For null hypothesis testing, the test statistic (e.g. *F*, *t*, *r*) with confidence intervals, effect sizes, degrees of freedom and *P* value noted<br>*Give P values as exact values whenever suitable.*
☐	☒	For Bayesian analysis, information on the choice of priors and Markov chain Monte Carlo settings
☐	☒	For hierarchical and complex designs, identification of the appropriate level for tests and full reporting of outcomes
☐	☒	Estimates of effect sizes (e.g. Cohen's *d*, Pearson's *r*), indicating how they were calculated

Our web collection on statistics for biologists contains articles on many of the points above.

Software and code

Policy information about availability of computer code

Data collection	No software was used.
Data analysis	R version 4.1.2; Stan version 2.21.3; NextClade 2.10.0; Illumina DRAGEN COVID Lineage Application 3.5.0, 3.5.1, 3.5.2, 3.5.3, 3.5.4; Pangolin 3.0. All code and data are available in a public GitHub repository: https://github.com/skissler/Ct_SequentialInfections

For manuscripts utilizing custom algorithms or software that are central to the research but not yet described in published literature, software must be made available to editors and reviewers. We strongly encourage code deposition in a community repository (e.g. GitHub). See the Nature Portfolio guidelines for submitting code & software for further information.

Data

Policy information about availability of data

All manuscripts must include a data availability statement. This statement should provide the following information, where applicable:

- Accession codes, unique identifiers, or web links for publicly available datasets
- A description of any restrictions on data availability
- For clinical datasets or third party data, please ensure that the statement adheres to our policy

All data are available in a public GitHub repository: https://github.com/skissler/Ct_SequentialInfections

Human research participants

Policy information about studies involving human research participants and Sex and Gender in Research.

Reporting on sex and gender	Data on sex/gender were not systematically collected nor used as a covariate in this study.
Population characteristics	The raw data consisted of 3,346 SARS-CoV-2 infections among 3,021 individuals, of which 1,989 infections were sufficiently well-documented to allow further analysis. Of these infections, 49 were caused by the alpha variant, 191 by the delta variant, 1400 by the BA.1/BA.2 variants, 71 by the BA.4/BA.5 variants, and 278 by other/unspecified variants. 816 infections were in individuals under the age of 30, 876 were in individuals aged 30-49, and 295 in individuals 50 and over.
Recruitment	The study population was a convenience sample of players, staff, and affiliates of the National Basketball Association. During the study period, testing was mandatory for players and staff and optional for other affiliates, biasing the sample demographics towards young, male participants. Informed consent for testing was obtained from all participants.
Ethics oversight	This work was approved as "research not involving human subjects" by the Yale Institutional Re-view Board (HIC protocol # 2000028599), as it involved de-identified samples. This work was al-so designated as "exempt" by the Harvard Institutional Review Board (IRB20-1407).

Note that full information on the approval of the study protocol must also be provided in the manuscript.

Field-specific reporting

Please select the one below that is the best fit for your research. If you are not sure, read the appropriate sections before making your selection.

☒ Life sciences ☐ Behavioural & social sciences ☐ Ecological, evolutionary & environmental sciences

For a reference copy of the document with all sections, see nature.com/documents/nr-reporting-summary-flat.pdf

Life sciences study design

All studies must disclose on these points even when the disclosure is negative.

Sample size	The study population was a convenience sample of 3,021 individuals infected with SARS-CoV-2. Infection with SARS-CoV-2 was a necessary criterion for inclusion. No sample size calculations were performed.
Data exclusions	We excluded 1,357 infections that were not sufficiently well documented to fit the viral kinetic statistical model (we required at least Ct < 40 and one Ct < 32). This left 1,989 infections.
Replication	Our central finding was a reduced viral clearance time associated with immunity from prior infection. While we could not conduct the study in other populations, we stratified the population in multiple ways to ensure robustness of our central findings. In particular, we assessed our main questions of (1) whether clearance time is reduced in second vs first infections and (2) whether the lineage of the first infection affects the kinetics of the second infection in a cohort of 71 individuals with a well-documented first and second infection, as well as in a larger cohort of 193 individuals with a well-documented second infection and any evidence of a prior infection.
Randomization	This was an observational study, and so no randomization occurred.
Blinding	The analysis was post-hoc, and so experimental groups were only assigned after all data had been collected. No blinding was performed because the study design was generated after all of the data had been collected.

Reporting for specific materials, systems and methods

We require information from authors about some types of materials, experimental systems and methods used in many studies. Here, indicate whether each material, system or method listed is relevant to your study. If you are not sure if a list item applies to your research, read the appropriate section before selecting a response.

Materials & experimental systems

n/a	Involved in the study
☒	☐ Antibodies
☒	☐ Eukaryotic cell lines
☒	☐ Palaeontology and archaeology
☒	☐ Animals and other organisms
☒	☐ Clinical data
☒	☐ Dual use research of concern

Methods

n/a	Involved in the study
☒	☐ ChIP-seq
☒	☐ Flow cytometry
☒	☐ MRI-based neuroimaging
